# 3-Dimensional objective aesthetic evaluation to replace panel assessment after breast-conserving treatment

**DOI:** 10.1007/s12282-020-01117-9

**Published:** 2020-06-19

**Authors:** Amy R. Godden, Rachel L. O’Connell, Peter A. Barry, Katherine C. D. Krupa, Lisa M. Wolf, Kabir Mohammed, Anna M. Kirby, Jennifer E. Rusby

**Affiliations:** 1 Royal Marsden Hospital, Downs Road, Sutton, Surrey, SM2 5PT UK; 2 Institute of Cancer Research, 15 Cotswold Road, Sutton, Surrey, SM2 5NG UK

**Keywords:** Breast, Cancer, Surgery, Conservation, Aesthetics, 3D-SI

## Abstract

**Background:**

Two-thirds of patients with early breast cancer undergo breast-conserving treatment (BCT). Aesthetic outcome is important and has long term implications for psychosocial wellbeing. The aesthetic goal of BCT is symmetry for which there is no gold-standard measure. Panel scoring is the most widely adopted assessment but has well-described limitations. This paper describes a model to objectively report aesthetic outcome using measures derived from 3-dimensional surface images (3D-SI).

**Method:**

Objective measures and panel assessment were undertaken independently for 3D-SI of women who underwent BCT 1–5 years previously. Univariate analysis was used to test for association between measures and panel score. A forward stepwise multiple linear regression model was fitted to identify 3D measurements that jointly predicted the mean panel score. The fitted model coefficients were used to predict mean panel scores for an independent validation set then compared to the mean observed panel score.

**Results:**

Very good intra-panel reliability was observed for the training and validation sets (wκ = 0.87, wκ = 0.84). Six 3D-measures were used in the multivariate model. There was a good correlation between the predicted and mean observed panel score in the training (*n* = 190) and validation (*n* = 100) sets (*r* = 0.68, *r* = 0.65). The 3D model tended to predict scores towards the median. The model was calibrated which improved the distribution of predicted scores.

**Conclusion:**

A six-variable objective aesthetic outcome model for BCT has been described and validated. This can predict and could replace panel assessment, facilitating the independent and unbiased evaluation of aesthetic outcome to communicate and compare results, benchmark practice, and raise standards.

## Introduction

Breast cancer is a common and emotive diagnosis with 54,722 new cases diagnosed in the UK in 2017 [1] Two-thirds of women managed surgically for breast cancer undergo Breast-Conserving Treatment (BCT). Aesthetic outcome after BCT has a well-documented influence on patients’ psychosocial wellbeing and quality of life [2–10]. With advancements in treatment and the excellent survival expectations of 90% at 1 year and 80% at 10 years [1], more women are living with the long-term impact of treatment. Surgeons and clinical oncologists should now focus on excellent long-term aesthetic outcome in addition to excellent disease control.

There is no gold standard measure for aesthetic outcome. Patient-Reported Outcome Measures (PROMs) have been used as an aesthetic evaluation method in their own right. However, PROMs lack objectivity and consistently report aesthetic outcome more favourably than panel assessment which highlights the need for an objective method of evaluation of aesthetics in addition to PROMs. Although anthropometric assessment, subjective rating scales, and photographic measurements have all been used to evaluate aesthetic outcome from breast surgery, none has been widely accepted and each comes with its own well-described limitations [11–18]. The intricacies of aesthetic evaluation are subtle and challenging to articulate and the complexities are reflected in poor agreement between patient, physician, and objective scales [9, 19, 20].

Panel assessment is the most widely accepted technique to measure aesthetic outcome in breast surgery but is inherently biased, costly, time-consuming, and un-standardised. The aesthetic goal of BCT is to achieve or maintain symmetry which is reflected in the most widely adopted scale, the Harvard Cosmesis Scale, developed by Harris et al. in the 1970s [21]. Panellists score symmetry between the breasts using a 4-point Likert scale from 1, which is poor, to 4, which is excellent. Deficiencies shared to a variable extent by all panel scales include lack of responsiveness (ability to distinguish clinically relevant differences), repeatability, and interpretability.

**Fig. 1:**
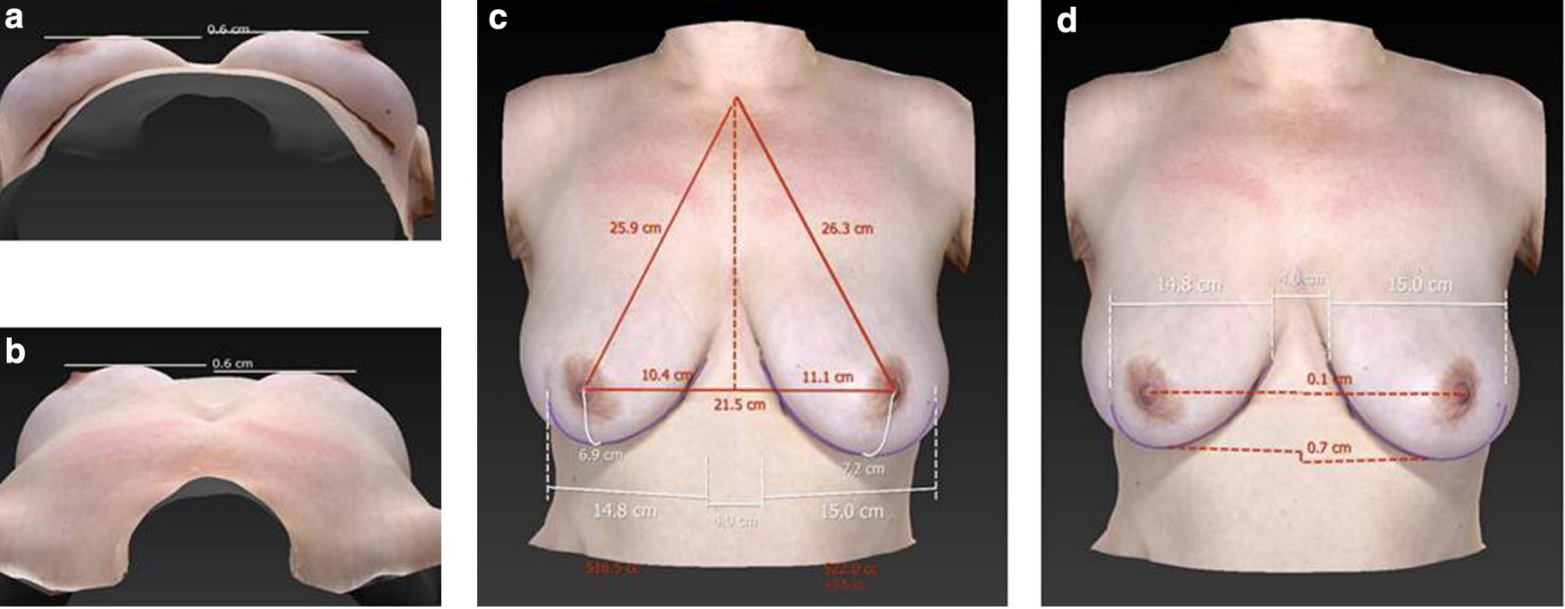
3D-SI in Mirror^®^ illustrating the cranial and caudal views (**a**, **b**) and linear measures (**a**–**d**)

3-Dimensional surface imaging (3D-SI) has the potential to overcome the limitations of alternative methods for evaluating aesthetics. It is simple to use and provides multiple views from one capture including the cranial and caudal views which help visualise projection and the infra-mammary fold (IMF) (Fig. 1). It delivers linear measures in addition to breast volume and surface symmetry calculations. These 3D-SI derived measures could replace panel assessment negating the subjective variability, inherent bias, and associated logistical challenges.

Objective evaluation of aesthetic outcome is essential for the communication and comparison of results e.g. between current and emerging techniques. It informs us of individual performance and can be used to benchmark performance between centres, regions, and at a national level. Robust reporting methods strengthen the evidence on which to base decisions and guidelines. The aim of this study was therefore to develop an objective aesthetic evaluation model based on measures derived from 3D-SI.

## Materials and methods

### Study design

The protocol was reviewed and approved by London-Riverside NRES committee [Ref 15/LO/0010] and is available at clinicaltrials.gov [NCT02304614]. The training set was recruited as part of an earlier study and an amendment was granted by NRES to analyse the images for the purpose of this study. The validation set was recruited independently using the same eligibility criteria.

Inclusion criteria were women who have had BCT for DCIS or invasive cancer within 1–6 years of study recruitment attending for surveillance mammography. Exclusion criteria included removal of the nipple-areola complex with no reconstruction, symmetrizing surgery, previous ipsilateral or contralateral breast surgery. Eligible potential participants were identified by working consecutively and chronologically through the surveillance mammography register.

Invitation to participate was by letter with a follow-up telephone call by a member of the study team to endorse the study. Participants had 3D-SI at the same time as their screening mammogram. The 3D-SIs were scored by an expert panel for the aesthetic outcome and objective measurements were performed independently as described in the sections below. Comparison between objective measures and panel score identified associations, and a model was built based on the relationships in a training set and validated using an independently recruited cohort from the same institution (validation set).

### Objective measures

The 3D-SIs were captured using VECTRA^®^ XT (Canfield Scientific) using a pre-defined protocol [22]. Objective measures were derived using Mirror^®^ software (Canfield Scientific). Validated methods were used to measure volume and surface symmetry which were calculated as an average of three measures [22]. The upper proportion was defined as the proportion of breast above the nipple. Independent measures e.g. Nipple to Sternal Notch (N–SN) distance were presented as the percentage difference between a patient’s breasts (%), and comparative measures e.g. surface asymmetry and projection, as absolute values.

### Panel assessment

The Panel comprised three consultant oncoplastic surgeons, a consultant radiation oncologist, and one senior breast care nurse. Panellists were blinded to the patient, operating surgeon and treating radiation oncologist identity. The Harvard cosmesis scale was used to assess AP, oblique, lateral, cranial and caudal views of 3D-SIs. The Harvard scale (1–4) is based upon symmetry: 1 = poor (treated breast seriously distorted), 2 = fair (treated breast clearly different from the untreated breast but not significantly distorted), 3 = good (treated breast slightly different from the untreated breast), and 4 = excellent (treated breast nearly identical to the untreated breast). The Likert scale was available throughout for reference. Individual panellist’s scores were recorded before a consensus panel score was agreed by discussion. The mean of the individual panellist’s scores was calculated for each image. Ten random images were presented more than once to test for internal consistency in the consensus scores for both the training and the validation set. The same panel was used to validate the model due to the inherent inconsistencies between panels rendering comparison between different panels unreliable. Examples of images from the training set receiving poor, fair, good and excellent scores were shown at the start of the assessment of the validation set to benchmark the panel.

### Statistical analysis

The training set was analysed using linear regression models to determine the relationship between each individual measurement and mean observed Harvard panel score. Then, a forward stepwise multiple linear regression model (at *p* < 0.05 variable inclusion) was fitted to identify the measurements that jointly predicted the mean observed Harvard panel score. The fitted model coefficients (intercept and slopes) were then used to predict panel scores for the validation dataset. The association between the mean observed and predicted panel score was assessed using scatter graphs and the correlation co-efficient (*r*) reported for both sets separately. Bland–Altman plots were used to assess agreement between mean observed and predicted panel scores and the mean difference and limits of agreement reported. Intra-panel agreement was assessed for repeated images and reported as weighted kappa (wκ) for both sets.

## Results

### Clinicopathological data

3D-SIs from 190 women were used for the training set and a further 100 women were recruited for the validation set. Clinico-pathological data for both sets were comparable (Table 1). Surgery was performed between 2009 and 2014 for the training set and 2010 and 2016 for the validation set. The median time (in months) from surgery to participation was 36 (IQR18-49) for the training set and 34 (IQR23-47) for the validation set. The tumour was located in the upper outer quadrant for the majority of women in both groups and most women had a standard wide local excision with no complex tissue rearrangement. All women in the training set and 94% of women in the validation set had adjuvant radiotherapy. The mean pre-operative tumour size (measured on ultrasound) for the training and validation sets was 14 mm and 16 mm, respectively. The median weight of the excision specimen was 32 g in the training set and 44 g in the validation set.

**Table 1:** Clinicopathological data for the training and validation sets

Clinico-pathological data	Training set *n* = 190	Validation set *n* = 100
Pre-operative data		
Age at time of surgery (years), mean (SD)	61 (11)	59 (11)
Time from surgery to study participation (months), median (IQR)	36 (18–49)	34 (23–47)
Ethnic origin		
White	178 (95)	91 (91)
Non-white	9 (5)	9 (9)
Smoking status (%)		
Never	114 (60)	58 (58)
Current	16 (8)	16 (16)
Ex-smoker	60 (32)	25 (25)
BMI at surgery (kg/m^2^), mean (SD)	27.42 (5.38)	27.51 (5.19)
Location of tumour on pre-operative imaging (%)		
Upper outer	104 (55)	50 (50)
Central	5 (3)	2 (2)
Lower inner	27 (14)	14 (14)
Lower outer	20 (11)	18 (18)
Upper inner	34 (18)	15 (15)
		1-unknown (external report)
US size (mm), mean (SD)	13.73 (8.58)	15.99 (8.54)
Mammographic size (mm), mean (SD)	16.08 (10.96)	18.16 (10.13)
Neoadjuvant therapy (%)		
None	167 (88)	92 (92)
Endocrine	9 (5)	2 (2)
Chemotherapy	14 (7)	6 (6)
Intra-operative data		
Experience of operating surgeon		
Consultant	105 (55)	47 (47)
Trainee with consultant scrubbed	41 (22)	17 (17)
Trainee with consultant un-scrubbed	44 (23)	36 (36)
Type of surgery (%)		
WLE	172 (91)	90 (90)
Other complex	18 (9)	10 (10)
Axillary surgery (%)		
Nil	16 (8)	10 (10)
SLNB or sampling	145 (76)	74 (74)
ALND	29 (16)	16 (16)
Re-excision of margins (%)		
No	160 (84)	88 (88)
Yes	30 (16)	12 (12)
Pathology data		
Tumour pathology size (mm), mean (SD)	21.51 (13.6)	24.02 (15.89)
Weight of specimen (g), median (IQR)	32 (20–48)	44 (22–59)
Tumour type on final pathology (%)		
IDC + DCIS	120 (63)	68 (68)
IDC	26 (14)	17 (17)
ILC	25 (13)	4 (4)
Other invasive	4 (2)	5 (5)
Total invasive	175 (92)	93 (93)
DCIS	15 (8)	6 (6)
Grade of invasive tumours (%)		
1	40 (23)	19 (20)
2	88 (50)	43 (47)
3	43 (25)	27 (29)
Not recorded	4 (2)	4 (4)
ER status of invasive tumours (%)		
Positive	157 (90)	82 (88)
Negative	18 (10)	11 (12)
PR status of invasive tumours (%)		
Positive	135 (77)	65 (70)
Negative	40 (23)	28 (30)
HER2 status of invasive tumours (%)		
Negative	165 (94)	87 (94)
Positive	9 (5)	6 (6)
Not recorded	1 (1)	
Triple negative tumours (%)	12 (7)	9 (10)
Nodal status (%)		
Negative	131 (69)	72 (72)
Positive	43 (23)	18 (18)
No axillary surgery	16 (8)	10 (10)
Adjuvant therapy		
Adjuvant chemotherapy (%)		
No	155 (82)	72 (72)
Yes	35 (18)	28 (28)
Adjuvant endocrine therapy (%)		
No	29 (15)	22 (22)
Yes	161 (85)	78 (78)
Adjuvant radiotherapy (%)		
No	0 (0)	6 (6)
Yes	190 (100)	94 (94)
Boost	50 (26)	28 (30)
SCF & axilla	11 (6)	7 (7)
Post-operative complications		
Delayed wound healing (> 30 days) (%)		
No	183 (95)	100 (100)
Yes	7 (5)	0 (0)

### Training set

Very good intra-panel consistency (wκ = 0.87) was observed for 10 repeated images in the training set, with 7/10 consensus scores agreeing and 3/10 varying by one point. In the validation set, the intra-panel agreement was also very good (wκ = 0.84) with 6/10 consensus scores agreeing and 4/10 varying by one point.

A significant relationship was identified between all but one (Nipple-to-Nipple distance) of the 3D-SI-derived measures and the mean panel score. Seven measures were found to be independently associated with mean panel score on multivariate analysis. Six of these variables were included in the multivariate model. The upper proportion difference produced similar measurements to Nipple-to-Sternal Notch (N-SN) distance and was considerably more time consuming to measure so was excluded. A summary of the variables is reported in Table 2.

**Table 2:** Univariate and multivariate analysis comparing 3D-SI measures with mean Harvard panel score

Variable	Constant (95% CI)	Coefficient (95% CI)	*p* value
**Univariate analysis**			
Upper proportion difference	3.21 (3.04–3.39)	− 0.059 (− 0.082: − 0.035)	< 0.001
N–M difference (%)	3.04 (2.87–3.21)	− 0.015 (− 0.027: − 0.003)	0.011
N–IMF difference (%)	3.09 (2.94–3.25)	− 0.014 (− 0.022: − 0.007)	< 0.001
N–SN difference (%)	3.38 (3.22–3.54)	− 0.079 (− 0.099: − 0.059)	< 0.001
Breast width difference (%)	3.07 (2.89–3.25)	− 0.043 (− 0.073: − 0.013)	0.005
M-MMF distance (cm)	2.62 (2.37–2.87)	0.097 (0.001: 0.184)	0.030
NH difference (cm)	3.31 (3.16–3.46)	− 0.256 (− 0.324: − 0.188)	< 0.001
IMF difference (cm)	3.30 (3.15–3.45)	− 0.355 (− 0.449: − 0.262)	< 0.001
Projection difference (cm)	3.08 (2.92–3.25)	− 0.344 (− 0.547: − 0.141)	0.001
N–N distance (cm)	3.74 (2.75–4.72)	− 0.036 (− 0.078: 0.005)	0.083
Volume symmetry (%)	1.22 (0.37–2.07)	0.019 (0.009: 0.029)	< 0.001
Surface asymmetry (mm)	3.87 (3.64–4.10)	− 0.156 (− 0.189: − 0.123)	< 0.001
**Multivariate analysis**			
Constant	3.137 (2.372: 3.902)	–	–
N–SN difference (%)	–	− 0.047 (− 0.068: − 0.026)	< 0.001
Breast width difference (%)	–	− 0.028 (− 0.052: − 0.004)	0.021
IMF difference (cm)	–	− 0.162 (− 0.267: − 0.057)	0.003
Projection difference (cm)	–	− 0.255 (− 0.424: − 0.086)	0.003
N–N distance (cm)	–	0.041 (0.007: 0.075)	0.017
Surface asymmetry (mm)	–	− 0.072 (− 0.116: − 0.028)	0.001

The model was built using forward stepwise multiple linear regression for the training set (at 5% alpha level) (*n* 190)

*RMS* root mean squared, *IMF* infra-mammary fold, *N–M* nipple–midline, *N–IMF* nipple-infra-mammary fold, *M–MMF* medial-medial mammary fold, *NH* nipple height, *N–N* nipple–nipple

A good correlation (*r* = 0.68) was seen between predicted and mean observed panel score for the training set. Bland–Altman analysis demonstrated a mean difference of 0 (95% CI − 0.084 to 0.084) between the observed panel score and the predicted score using the 3D model suggesting no bias, with narrow limits of agreement within which 95% of the differences fall (− 1.173 to 1.173).

### Validation set

A summary of the mean observed Harvard panel score, predicted panel score (using the multivariate model), and 3D-SI measures for the training and validation set are presented in Table 3. A good correlation was found between the predicted and mean observed panel score for the validation set (*r* = 0.65). Bland–Altman analysis demonstrates a mean difference of − 0.055 (95% CI − 0.166: 0.056) between the observed panel score and the predicted score using the 3D model suggesting no bias, with narrow limits of agreement within which 95% of the differences fall (− 1.173 to 1.062).

**Table 3:** 3D-SI measures for the training and validated sets and a summary of the mean observed Harvard panel scores and the predicted panel score using the multivariate model

	Training set *n* = 190 Mean (SD)	Validation set *n* = 100 Mean (SD)	
Measures from 3D-SI			
Surface asymmetry (mm)	6.40 (2.86)	7.11 (2.97)	
NSN difference (%)	6.47 (4.97)	5.44 (4.35)	
IMF height difference (cm)	1.21 (1.07)	1.12 (1.03)	
Projection difference (cm)	0.61 (0.54)	0.61 (0.52)	
N–N distance (%)	23.76 (2.74)	23.99 (2.80)	
Breast width difference (%)	4.62 (3.72)	5.31 (3.42)	

Harvard panel score	Observed score	Observed score	Predicted score for validation set
Median	3	3	3
Range	1–4	1–4	1–4
IQR	2–3.6	2.2–3.6	2.57–3.25
Mean (SD)	2.87 (0.79)	2.93 (0.78)	2.87 (0.54)

### Calibrated model

**Fig. 2:**
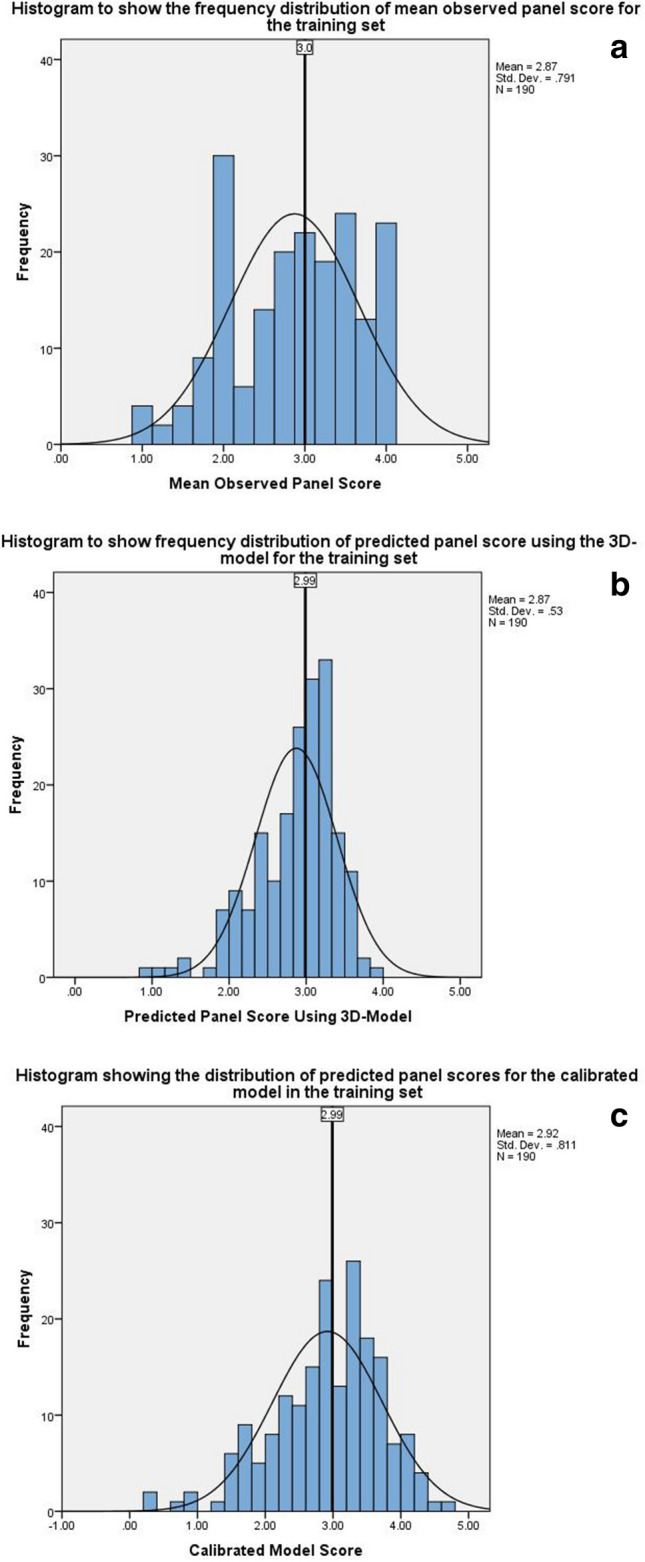
Histograms to show the frequency distribution of the mean observed Harvard panel scores (**a**), 3D model (**b**), and the calibrated model (**c**) for the training set (*n* = 190)

Bland–Altman analysis illustrated that the 3D model over-predicts for lower panel scores, and under predicts for higher panel scores. Histograms corroborate this finding by illustrating a clustering of predicted scores about the median (Fig. 2b). To improve the spread of predicted scores, the model was calibrated to the mean observed frequency distribution of panel score in the training set.

**Fig. 3:**
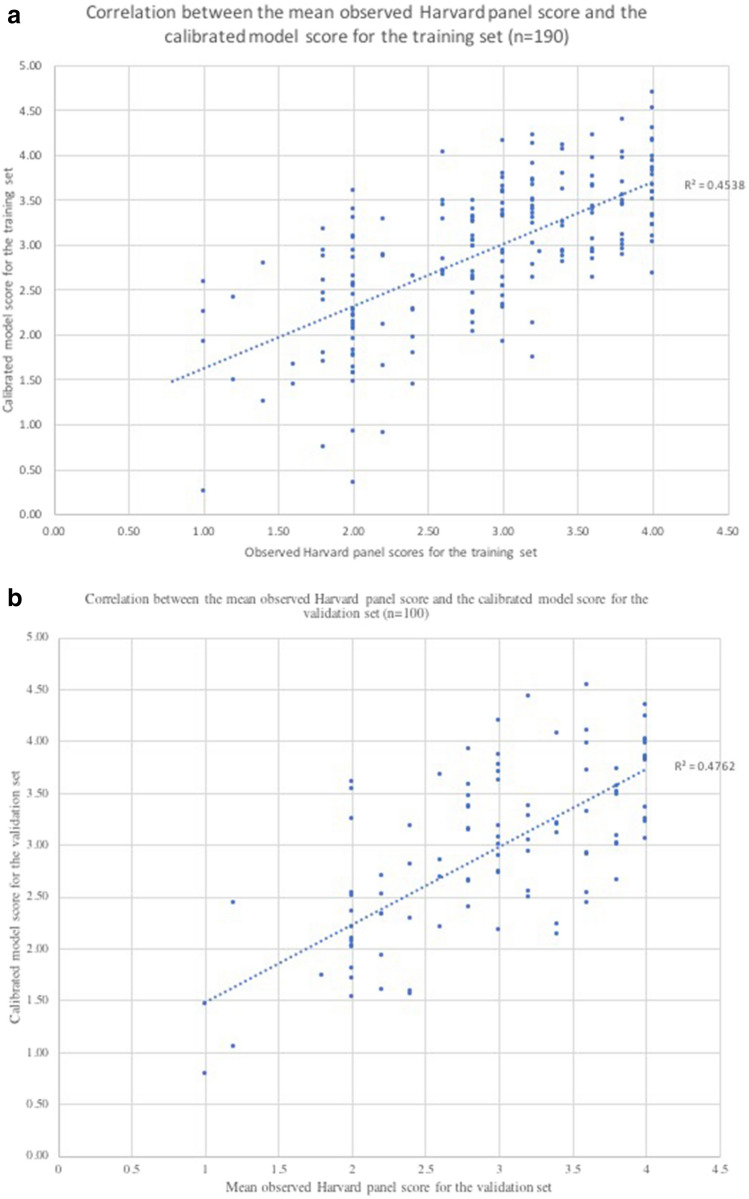
Scatter plots illustrating the correlation between the observed Harvard panel score and the Calibrated Model for the training set (**a**) and the validation set (**b**). Correlation co-efficient *r* = 0.67 and 0.69 respectively

The correlation between the calibrated model and the mean observed panel scores is similar to that of the 3D model (*r* = 0.67 and 0.69 for the training and validation sets, respectively) (Fig. 3a, b). Bland–Altman analysis of the calibrated model demonstrated a mean difference of − 0.05 and 0 for the training and validation sets compared to the calibrated model respectively, suggesting no bias, with narrow limits of agreement within which 95% of the differences fall (− 1.32 to 1.23 for the training set and − 1.27 to 1.28 for the validation set). Histograms demonstrate the improved distribution of scores for the calibrated model compared to the 3D model with reference to the distribution of the mean observed panel score (Fig. 2). This is reflected in the broader IQR observed in the calibrated model versus 3D model in Table 4. The net result is a model that has a very similar correlation and agreement with the observed panel score, with more discrimination between outcomes i.e. scores are not clustered at the median value.

**Table 4:** Descriptive statistics for the mean observed panel scores, 3D Model, and calibrated model for the training and validation sets

	Observed		3D-Model		Calibrated Model	
	Training	Validation	Training	Validation	Training	Validation
Min	1	1	0.96	0.75	0.25	− 0.04
Max	4	4	3.85	3.77	4.69	4.54
Mean	2.87	2.93	2.87	2.87	2.92	2.93
SD	0.79	0.78	0.53	0.55	0.81	0.85
Median	3.0	3.0	2.99	3.02	2.98	3.03
Quartile 1	2	2.2	2.56	2.54	2.44	2.38
Quartile 3	3.6	3.6	3.24	3.29	3.49	3.57

**Fig. 4:**
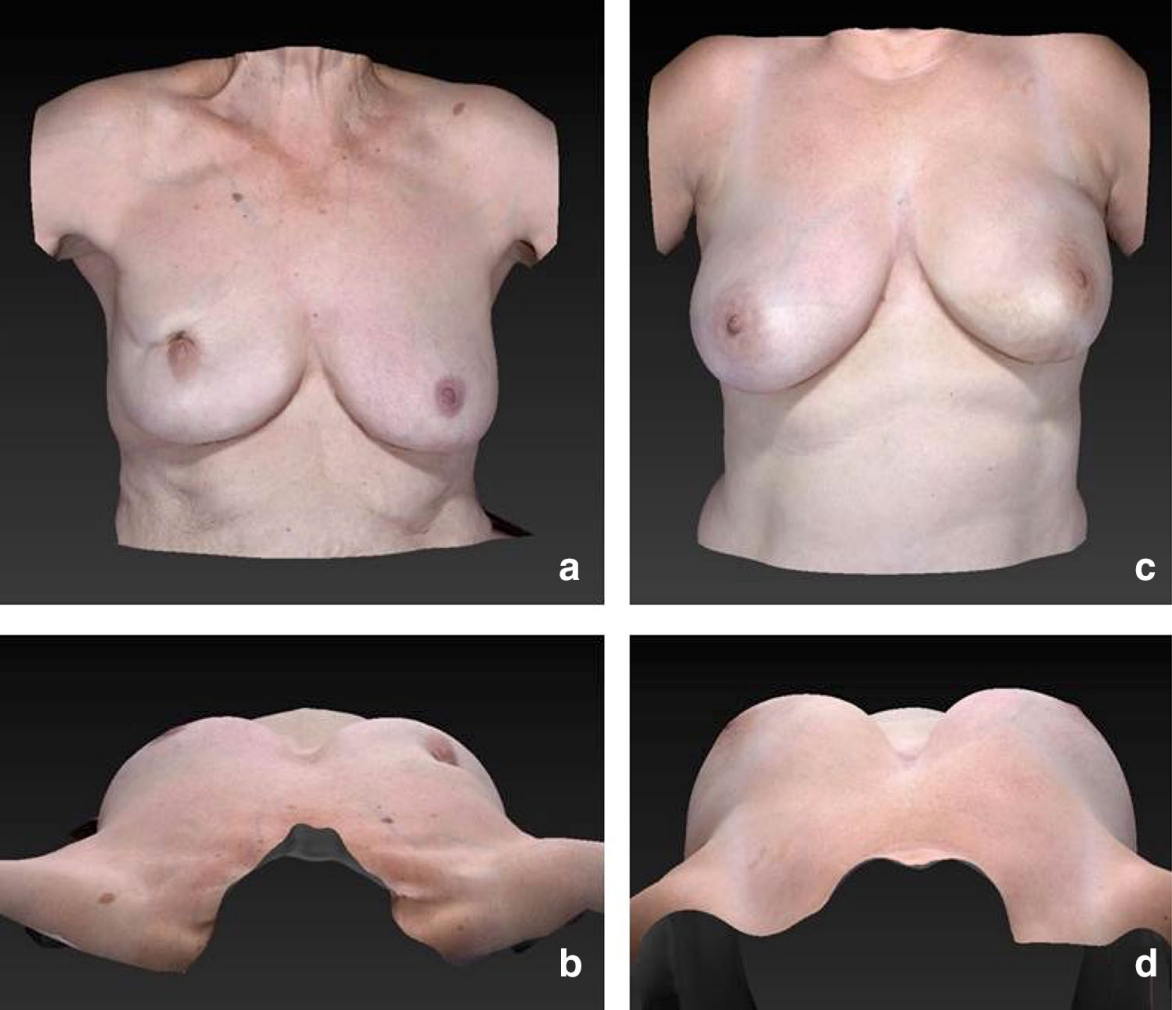
Left; observed Harvard panel score of 1.4 and 3D-model score of 2.8. A focal deficit in the upper outer breast detracts from the overall aesthetic result, however, may not be captured in the overall asymmetry score (rms) delivered by 3D-SI analysis (**a**, **b**). Right; observed Harvard panel score of 2.4 and 3D-model score of 2.3. Global volume and surface asymmetry between operated and non-operated breast are accurately detected by 3D-SI analysis (**c**, **d**)

In the training set, the calibrated model correctly predicted panel score to within 0.5 points of the mean observed Harvard panel score in 99 (52%), within 1 point in 166 (87%), within 1.5 points in 187 (98%) and all patients within 2 points. In the validation set the calibrated model correctly predicted panel score to within 0.5 points of the mean observed Harvard panel score in 57 (57%), within 1 point in 86 (86%), within 1.5 points in 97 (97%) and all patients within 2 points. In-depth analysis of cases where the model over predicted by more than 1.5 points illustrated focal volume deficits which detract from the overall aesthetic result which may not have been captured by the overall asymmetry score delivered during 3D-SI analysis (Fig. 4).

## Discussion

This paper describes the development of a six-variable objective aesthetic outcome model for Breast-Conserving Treatment (BCT) which can predict and could ultimately replace panel assessment. The model accurately measures and reports aesthetic outcome incorporating evaluation of views unique to three-dimensional photography enabling surface symmetry and projection to be incorporated into the assessment, a potential advantage over 2D images.

Many attempts have been made to objectively evaluate the aesthetic outcome of breast surgery, however, each method has its limitations [23, 24]. The Breast Cancer Conservative Treatment. cosmetic results (BCCT.core) model is the most widely cited in the literature [25–28]. The BCCT.core model evaluates breast asymmetry in two dimensions so measures such as volume, 3D surface symmetry, and projection cannot be evaluated. The breast is a 3-dimensional structure, therefore, is not comprehensively assessed in two dimensions. 3D-SI has the ability to produce volume and shape symmetry measures which have recently been validated in-vivo providing an additional component to objective aesthetic evaluation [22].

Cardoso et al. have recently published results for a 3D version of the BCCT.core model based on the capabilities of Microsoft Kinect. They concluded the addition of the third dimension is not necessary, based on the lack of improvement in the association between model and panel score [29]. The conclusion was based on the addition of a single 3D parameter to BCCT.core, volume symmetry, which was not found to be independently associated with panel score on multivariate analysis in our study. Additional capabilities of 3D measures were not included, such as surface symmetry and projection, so the conclusion was perhaps drawn upon an oversimplified application of 3D technology. Another advantage of the 3D model described in this paper is that it produces a score on a continuous scale, enabling more detailed feedback on performance i.e. a score of 2.4 or 1.5 would be delivered rather than a score of 2, which would be the rounded score for both.

Clinicians and patients may have divergent views of what constitutes a good aesthetic outcome. Potter et al. outlined a core outcome set for breast reconstruction based on Delphi methodology in which ‘patient satisfaction with cosmetic outcome’ was rated highly amongst medical professionals and patients alike [30]. Patient-Reported Outcome Measures (PROMs) are clearly the most important evaluation of aesthetic outcome but lack objectivity, are affected by the treatment path leading to the final outcome and are consistently discordant from professional assessment, being frequently reported more favourably [9, 31–33]. Dahlbäck et al. have recently emphasised the importance of PROMs in aesthetic evaluation demonstrating a stronger predictive ability for longer-term health-related quality of life as compared to objective measures or panel assessment [9]. The objective model described in this paper is not designed to replace PROMs, and PROMs cannot obviate the need for an objective model designed to produce an independent and unbiased evaluation of aesthetic outcome. The two methods of aesthetic evaluation must co-exist, and development into a combined outcome set for BCT may be considered a further area of study.

A very good intra-panel agreement using the Harvard scale (wκ = 0.87, wκ = 0.84 for test and validation sets respectively) is reported. However, the reported internal consistency of panel assessment is variable in the literature illustrating one of the limitations of this evaluation method [9, 26, 32, 34, 35]. Even when panellists were selected from a group of experts based upon the agreement of their previous scores with the consensus opinion, their individual Harvard score switched category to match consensus 30% of the time [27]. The logistics of arranging a panel assessment are complex and inefficient both in terms of time and cost. Objective assessment using 3D-SI can be performed on a case by case basis with greater flexibility and a greatly reduced time and resource burden.

The surface asymmetry measure in Mirror^®^ gives an average over the entire breast surface (root mean squared), thereby giving a representative result when there is global surface asymmetry or surface asymmetry affecting a moderate area of the breast. However, for very small areas of volume deficit in an otherwise symmetrical breast, the focal surface asymmetry will be countered by the remaining global surface symmetry, so can be ‘hidden’ in the measure. The ability measure and report upon a focal volume deficit is an area for development which may help to refine the accuracy of the model in the small subset of patients affected by this.

To improve the applicability into everyday practice, the software requires development to enable automated calculation of the outcome score. In addition, there is some difficulty imaging women with very large volume breasts and on occasion the lateral view is cropped to the mid-axillary line to enable capture of the anterior contour of the breasts. The automatic placement of surface landmarks is less reliable for larger breasts and moderate ptosis, sometimes requiring manual adjustment or placement, which decreases the efficiency of measuring. However, manually placing landmarks is still very quick and the software provides diagrams to guide placement so prior training is not essential.

The model was based upon and tested against a clearly defined method of expert panel assessment with very-good internal consistency, a large dataset of 3D-SIs and included an independently recruited cohort for validation. Validation at a different centre, or within a prospectively-collected cohort is an area for future work. A prospective study would also eliminate selection bias. For now, it is encouraging that the median Q-score for “satisfaction with breasts “ for the training set using the BREAST-Q BCT module was 68 (IQR 55–80) out of 100, where 100 is best. This is concordant with other contemporary analyses where the median *Q*-scores 3–6 years after surgery ranged from 65 to 68 [9].

It may be possible to extend the principle used within this study to women who have undergone breast reconstruction, however, a large multicenter study would be required to generate a 3D-SI library large enough to reflect the diversity in practice in the UK. Survivorship is a rapidly expanding area of interest, and continued development of portable, cheaper 3D capture systems has the potential to revolutionise aesthetic evaluation by the integration of 3D-SI into research and clinical practice.
